# The Kinase Activity of Calcineurin B-like Interacting Protein Kinase 26 (CIPK26) Influences Its Own Stability and that of the ABA-regulated Ubiquitin Ligase, Keep on Going (KEG)

**DOI:** 10.3389/fpls.2017.00502

**Published:** 2017-04-10

**Authors:** Wendy J. Lyzenga, Victoria Sullivan, Hongxia Liu, Sophia L. Stone

**Affiliations:** Department of Biology, Dalhousie University, HalifaxNS, Canada

**Keywords:** ABA, CIPK26, RING-type E3 ligase, KEG, ubiquitination, 26S proteasome, protein degradation, phosphorylation

## Abstract

The Really Interesting New Gene (RING)-type E3 ligase, Keep on Going (KEG) plays a critical role in Arabidopsis growth after germination and the connections between KEG and hormone signaling pathways are expanding. With regards to abscisic acid (ABA) signaling, KEG targets ABA-responsive transcription factors abscisic acid insensitive 5, ABF1 and ABF3 for ubiquitination and subsequent degradation through the 26S proteasome. Regulation of E3 ligases through self-ubiquitination is common to RING-type E3 ligases and ABA promotes KEG self-ubiquitination and degradation. ABA-mediated degradation of KEG is phosphorylation-dependent; however, upstream signaling proteins that may regulate KEG stability have not been characterized. In this report, we show that CBL-Interacting Protein Kinase (CIPK) 26 can phosphorylate KEG *in vitro.* Using both *in vitro* and *in planta* degradation assays we provide evidence which suggests that the kinase activity of CIPK26 promotes the degradation of KEG. Furthermore, we found that the kinase activity of CIPK26 also influences its own stability; a constitutively active version is more stable than a wild type or a kinase dead version. Our results suggest a reciprocal regulation model wherein an activated and stable CIPK26 phosphorylates KEG to promote degradation of the E3.

## Introduction

The ubiquitin proteasome system (UPS) is responsible for the degradation of numerous proteins and regulates a wide range of cellular events. Ubiquitin-dependent proteolysis plays an indispensable role in the regulation of plant hormone production, perception, signal transduction and output (Santner and Estelle, 2010; Liu and Stone, 2011; Lyzenga and Stone, 2012). Central to the ubiquitination pathway are the ubiquitin ligases (E3) that govern target selection. They facilitate transfer of ubiquitin molecules onto the selected protein prior to degradation by the 26S proteasome. Keep on Going (KEG) is a single subunit Really Interesting New Gene (RING)-type E3 that plays an essential role during early plant development. *KEG* mutant seeds germinate but fail to develop beyond the early seedling stage (Stone et al., 2006). KEG is a large protein consisting of two catalytic domains, RING and kinase domains, and two series of repeats, ankyrin and HERC2-like, used for protein-protein interaction. KEG’s role during plant development appears to be multifaceted as evidence suggests that KEG is a regulator of both abscisic acid (ABA) and jasmonate (JA) signaling (Stone et al., 2006; Liu and Stone, 2010, 2013; Chen et al., 2013; Lyzenga et al., 2013; Pauwels et al., 2015), in addition to regulation of post-golgi trafficking (Gu and Innes, 2012).

Abscisic acid acts through a multilayered signaling cascade that culminates in the activation of various transcription factors and changes in the expression of genes required for responses to stress or developmental transitions. In the absence of ABA, KEG negatively regulates the abundance of members of the bZIP subfamily of transcription factors such as Abscisic Acid Insensitive 5 (ABI5), Abscisic Acid Responsive Element-Binding Factor 1 (ABF1) and ABF3 (Stone et al., 2006; Liu and Stone, 2010; Chen et al., 2013). Loss of *ABI5*, *ABF1* or *ABF3* in the *keg-1* background is able to partially rescue the severe early growth arrest of *keg* seedlings, confirming that mis-regulation of ABA signaling contributes to the mutant phenotype (Stone et al., 2006; Chen et al., 2013). However, lack of a complete rescue suggests that KEG has other targets. A member of the CBL-Interacting Protein kinase (CIPK) family, CIPK26, was also identified as a KEG-interacting protein (Lyzenga et al., 2013; Pauwels et al., 2015). CIPK26 is an ubiquitination substrate and KEG targets both the kinase CIPK26 and downstream transcription factors (e.g., ABI5) for degradation by the 26S proteasome (Lyzenga et al., 2013).

The CIPK family of kinases are also known as Sucrose Non-Fermenting (SNF)-related Kinase 3 (SnRK3s) and belong to the SNF1-related kinases along with the SnRK1 and SnRK2 subfamilies (Hrabak et al., 2003). CIPKs have been shown to regulate ABA signaling, abiotic stress response and facilitate ion homeostasis through regulation of various ion transporters (Yu et al., 2014). Interestingly, CIPK26 was recently shown to interact with the SnRK2, SRK2D/SnRK2.2, and the two kinases facilitate Mg^2+^ homeostasis possibly through a basal level ABA-related mechanism (Mogami et al., 2015).

The interaction between KEG and CIPK26 likely acts as part of the ABA signaling network as CIPK26 can interact with upstream phosphatases ABI1/2 and phosphorylate ABI5 (Lyzenga et al., 2013). ABA regulates KEG’s E3 ligase activity by promoting KEG’s self-ubiquitination and reducing KEG protein levels. Previous work has shown that phosphorylation is required for ABA-dependent KEG self-ubiquitination and degradation (Liu and Stone, 2010). However, kinases that influence KEG’s stability have not been characterized. In this report we investigated the possibility of reciprocal regulation between CIPK26 and KEG. We found that CIPK26 can phosphorylate KEG *in vitro* and using both cell-free and *in planta* degradation assays we found that an active version of CIPK26 can promote the degradation KEG. Moreover, we found that the kinase activity of CIPK26 influences its own stability; a constitutively active version of the kinase is more stable than wild type and an inactive version of the kinase is highly unstable. Our results support a model wherein an activated and stable CIPK26 phosphorylates KEG to promote degradation of the E3.

## Materials and Methods

### Cloning and Mutagenesis

Gateway cloning was used to generate all constructs unless indicated (Invitrogen). Constructs relating to full length CIPK26, partial CIPK26N, and kinase variants have been previously reported (Lyzenga et al., 2013). Constructs relating to KEG have been described previously (Liu and Stone, 2010). In addition, full length CIPK26, CIPK26^TD^ or CIPK26^KR^ cDNA was introduced into the pEarleyGate101 Gateway plant transformation vector for expression of C-terminal tagged yellow fluorescence protein (YFP) and hemagglutinin (HA) protein under control of the cauliflower mosaic virus 35S promoter (Earley et al., 2006). The 17-β-estradiol-inducible expression vector was generated as previously described (Lyzenga et al., 2013). CIPK26-YFP, CIPK26^TD^-YFP, and CIPK26^KR^-YFP cDNA was PCR amplified from the corresponding pEarleyGate101 constructs using primers listed below and then cloned into pDONR201 and subsequently introduced into the 17-β-estradiol-inducible vector to create *35S:XVE/OlexA:CIPK26-YFP-HA*, *35S:XVE/OlexA:CIPK26^TD^-YFP-HA*, and *35S:XVE/OlexA:CIPK26^KR^-YFP-HA* (referred to as *OlexA:CIPK26-YFP-HA*, *OlexA:CIPK26^TD^-YFP-HA*, and *OlexA:CIPK26^KR^-YFP-HA* in this report). Primers used in this study: CIPK26-YFP Forward, 5′GGGGACAAGTTTGTACAAAAAAGCAGGCTTCATGAGTAAAGGAGAAGAACTTTTCACTGG-3′; CIPK26-YFP Reverse, 5′-GGGGACCACTTTGTACAAGAAAGCTGGGTCAGCGTAATCTGGAACATCGTATGGG-3′.

### Plant Material and Growth Conditions

*Arabidopsis thaliana* ecotype Columbia (Col-0) wild type and transgenic seeds were surface-sterilized with 50% (v/v) bleach and 0.1% Triton X-100. After cold treatment at 4°C for 2 days, seeds were germinated and grown on half strength solid Murashige and Skoog (MS) medium containing 0.8 agar and 1% sucrose under continuous light at 22°C. For plants grown in soil, 7-day-old seedlings were transferred from solid MS medium to soil and grown under photoperiodic cycles of 16 h light and 8 h dark at 22°C. For inhibitor assays, 4- or 6-day-old seedlings were transferred from solid MS to liquid MS medium and grown under continuous light at 22°C.

### Plant Transformation

All constructs were introduced into *Agrobacterium tumefaciens* strain GV3101 and transgenic Arabidopsis plants were generated using the floral dip method (Clough and Bent, 1998). In order to generate double-transgenic plants, a previously generated *35S:HA-KEG/keg-1* line was transformed with *OlexA:CIPK26-YFP-HA, OlexA:CIPK26^TD^-YFP-HA*, or *OlexA:CIPK26^KR^-YFP-HA* (Liu and Stone, 2010). Transgenic plants were selected by growing seedlings on half-strength solid MS medium plus supplemented with the appropriate herbicide selection. Resistant transformants were transferred to soil and allowed to set seeds. The presence of each transgene was verified by genotyping using polymerase chain reaction (PCR) and protein expression was determined by western blot analysis.

### Protein Extraction and Western Blot Analysis

For all experiments plant tissue was snap frozen in liquid nitrogen and ground to a fine power before addition of protein extraction buffer [20 mM Tris-HCl, pH 7.5, 150 mM NaCl, 1 mM EDTA, 5% glycerol, and protease inhibitor cocktail tablets (Roche Diagnostics)]. Depending on the assay between 30 and 50 ug of protein from each treatment was subject to western blot analysis. Antibodies were used according to manufactures specifications (mouse anti-HA 1:10,000; rabbit anti-GFP 1:5,000; anti-mouse IgG 1:10,000; anti-rabbit IgG 1:5,000) and are all from Sigma. All assays were repeated at least twice.

### MG132, 17-β-estradiol, CHX, and ABA Treatment Assays

To assess the effect of proteasome inhibitor (MG132) on protein accumulation, transiently transformed *Nicotiana benthamiana* (tobacco) leaves expressing GFP-CIPK26, GFP-CIPK26^TD^ or GFP-CIPK26^KR^ were injected with 50 μM MG132 24 h after *Agrobacterium* infiltration, excised, and incubated in 50 μM MG132 for another 16 h before tissue was analyzed for protein expression. Transient protein expression in tobacco was carried out as previously described (Liu et al., 2012).

Four-day-old 35S:CIPK26-YFP-HA, 35S:CIPK26^TD^-YFP-HA, 35S:CIPK26^KR^-YFP-HA transgenic seedlings were transferred to liquid MS medium and treated with 30 μM MG132 or DMSO (control) for 16 h then collected at the indicated time points and analyzed for protein expression.

To demonstrate the effect of activated CIPK26-YFP-HA on HA-KEG protein abundance in a cell-free degradation assay, 6-day-old *OlexA:CIPK26^TD^-YFP-HA, 35S:HA-KEG/keg-1* and *OlexA:CIPK26^KR^-YFP-HA, 35S:HA-KEG/keg-1* double transgenic seedlings treated with 20 μM 17-β-estradiol for 4 h before tissue collection in liquid nitrogen.

To demonstrate the effect of activated CIPK26-YFP-HA on HA-KEG protein abundance over time *in planta*, 4-day-old*OLexA:CIPK26^TD^-YFP-HA, 35S:HA-KEG/keg-1* and *OLexA: CIPK26^KR^-YFP-HA, 35S:HA-KEG/keg-1* double transgenic seedlings were grown for 24 h in liquid MS medium and then treated with 20 μM 17-β-estradiol or with ethanol (solvent, control) for 6 h. Seedlings were then treated with 500 μM cycloheximide (CHX), an inhibitor of protein synthesis, and tissue was collected at specified time points.

### Purification of Recombinant Proteins

Recombinant proteins were expressed in *Escherichia coli* strain Rosetta (DE3) and purified using nickel-charged resin (Bio-Rad) according to the manufacturer’s protocols.

### Cell-Free Degradation Assays

Cell-free degradation assays were adapted from a previous report (Wang et al., 2009). In brief, tissue was collected from an appropriate treatment and ground in protein extraction buffer. At time zero, 10 mM MgCl_2_ and 10 mM ATP was added to between 500 ng and 1 mg of total plant protein extract and the reactions were incubated at 25°C. Equal volume of sample was taken at the indicated time points and the reaction was stopped by the addition of SDS-loading buffer. A modified degradation assays used 100ng Flag-His-CIPK26N^TD^ or Flag-His-CIPK26N^KR^ recombinant proteins added at time zero to the reaction and then incubated at 30°C. For proteasome inhibitor treatment, 50 μM MG132 was added to the total protein extract 30 min before time zero and the addition of ATP, MgCl_2_ and recombinant proteins.

### Kinase Assay

Combinations of wild type and mutated forms of recombinant His-Flag-CIPK26N and His-Flag-KEGRK were incubated at 30°C for 30 min in 30 μL kinase assay buffer adapted from previously described (20 mM Tris-HCl, pH 7.5, 50 mM NaCl, 1 mM DTT, 0.1% Triton X-100, and 10 μCi of [γ-33P]ATP) (Ren et al., 2002). The reaction was stopped by the addition of SDS-loading buffer and boiling for 5 min. Samples were separated on a 7.5% SDS-PAGE gel and the gel was dried with a gel dryer (Biorad) and phosphorylated protein was detected by autoradiography. Kinase assay was repeated twice.

### Sequence Alignment

KEG amino acid sequences were aligned using CLC sequence viewer 7.7.1^1^. Amino acid sequences were retrieved from National Center for Biotechnology Information (NCBI) and the accession numbers are as follows: *Camelina sativa*: XP_010492113.1; *Capsella rubella*: XP_006289271.1; *Brassica napus*: XP_013728211.1; *Populus trichocarpa*: XP_006368632.1; *Gossypium arboreum*: XP_017615233.1; *Glycine max*: XP_003538267.1; *Medicago truncatula*: XP_003598471.2; *Oryza sativa*: EEC_79164; *Sorghum bicolor*: XP_002441049.

## Results

### CIPK26 Phosphorylates KEG *In vitro* and an Active CIPK26 Promotes KEG Degradation *In vitro* and *In vivo*

Previous work has shown that phosphorylation is involved in the ABA-mediated self-ubiquitination and degradation of KEG (Liu and Stone, 2010). Searches of the Arabidopsis Protein Phosphorylation Site Database (PhosPhAt 4.0) and Plant Protein Phosphorylation Database (P3DB) identified five (S195, S438, S436, S440, and S974) experimentally determined phosphorylation sites within KEG (Gao et al., 2009; Durek et al., 2010; Yao et al., 2012, 2014; **Supplementary Figure S1A**). To determine if these amino acids may represent relevant and conserved phosphorylation sites we compared KEG amino acid sequence to those from nine other plant species (**Supplementary File S1** and **Figures S1B**, **2**). Two of the five phosphorylation sites were conserved across the Brassicacea family (S195 and S438), while the others were conserved across all species examined (S436, S440, and S974).

**FIGURE 1:**
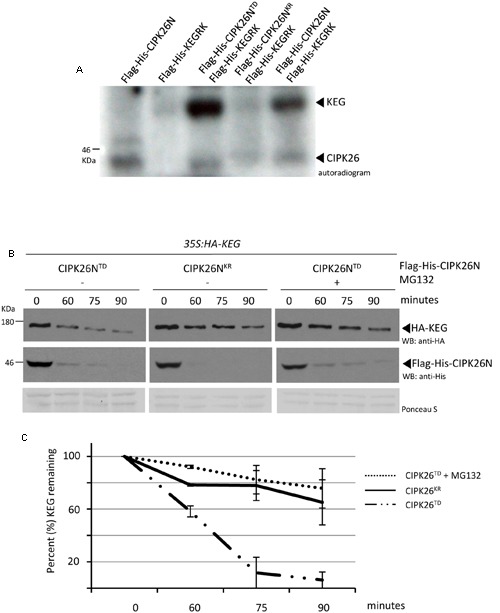
**Calcineurin B-like interacting protein kinase 26 (CIPK26) can phosphorylate KEG *in vitro* and active CIPK26 promotes the proteasome-dependent turnover of KEG in a cell free degradation assay. (A)** *In vitro* phosphorylation assays using Flag-His-KEGRK and Flag-His-CIPK26N, Flag-His-CIPK26N^KR^ (inactive) or Flag-His-CIPK26N^TD^ (constitutively active). Autoradiogram shows self-phosphorylation of CIPK26N and phosphorylation of KEG by CIPK26N and CIPK26N^TD^. Autoradiogram is representative of two trials. **(B)** Modified cell free degradation assay in which purified Flag-His-CIPK26N^TD^ or Flag-His-CIPK26N^KR^ was added to protein extracts prepared from 6-day-old *35S:HA-KEG* seedlings. HA-KEG protein levels were determined by western blotting (WB) using HA antibodies at the indicated time points. **(C)** Quantification of two trials using signal intensity at each time point determined by IMAGEJ. The error bar represents ± standard error.

In an attempt to identify the kinase involved in KEG phosphorylation, we examined the ability of the CIPK26, a known interactor (Lyzenga et al., 2013; Pauwels et al., 2015), to directly phosphorylate the E3. Phosphorylation assays were carried out using the N-terminal kinase domain of CIPK26 (Flag-His-CIPK26N) and the RING and kinase region of KEG (Flag-His-KEGRK). Full-length CIPK26 is insoluble and we were not able to purify sufficient protein for the assay. Likewise, the large size of the KEG protein (178KDa) hinders purification, therefore only the RING and kinase segment of KEG was used in the assay. We found that His-Flag-CIPK26N displayed self-phosphorylation and was able to phosphorylate the His-Flag-KEGRK (**Figure 1A**). The constitutively active version of the kinase (His-Flag-CIPK26N^TD^) was also able to efficiently phosphorylate Flag-His-KEGRK. Phosphorylation of KEG was not observed when the inactive version of CIPK26N (Flag-His-CIPK26N^KR^) was used in the assay (**Figure 1A**). These results show that CIPK26 can phosphorylate KEG *in vitro*.

Since general kinase activity is required for ABA-induced KEG degradation we next sought to determine if CIPK26 is involved in regulating KEG abundance (Liu and Stone, 2010). We first used a modified cell-free degradation assay that utilized a purified recombinant kinase and protein extract from transgenic seedlings expressing HA-KEG. Recombinant His-Flag-CIPK26N^TD^ or His-Flag-CIPK26N^KR^ was added to protein extract prepared from 6-day-old *35S:HA-KEG/keg-1* transgenic Arabidopsis seedlings and samples were collected at the time points indicated (**Figure 1B**). The abundance of HA-KEG was found to markedly decrease in the presence of activated CIPK26 (Flag-His-CIPK26N^TD^) compared to the slight decrease observed in the presence of the inactive kinase (Flag-His-CIPK26N^KR^) (**Figures 1B,C** and **Supplementary Figure S3A**). The decrease in HA-KEG protein levels in the presence of Flag-His-CIPK26N^TD^ was not observed when proteasome inhibitor, MG132, was included in the assay (**Figures 1B,C**).

**FIGURE 2:**
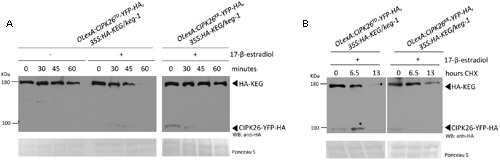
**Activated CIPK26 promotes the degradation of KEG *in planta*. (A)** Cell-free degradation assay using protein extracts from 5-day-old *OLexA:CIPK26^TD^-YFP-HA, 35S:HA-KEG/keg-1* (line 1) and *OLexA:CIPK26^KR^-YFP-HA, 35S:HA-KEG/keg*-1 (line 1) seedlings induced to express CIPK26 with 20 μM 17-β-estradiol. HA-KEG protein abundance was determined by WB using HA antibodies at the indicated time points. **(B)** Cycloheximide (CHX) chase assay using 5-day-old *OLexA:CIPK26^TD^-YFP-HA, 35S:HA-KEG/keg-1* (line 1) and *OLexA:CIPK26 ^KR^-YFP-HA, 35S:HA-KEG/keg-1* (line 1). Seedlings were incubated in liquid growth medium supplemented with or without 20 μM 17-β-estradiol to induce expression of CIPK26. Seedlings were then treated with CHX and samples collected at the indicated time points. The abundance of HA-KEG present at each time point was determined by WB with HA antibodies.

To determine if CIPK26 is involved in regulating KEG abundance *in planta*, independent double-transgenic Arabidopsis *keg-1* plant lines expressing HA-KEG (*35S:HA-KEG/keg-1*) and active CIPK26^TD^-YFP-HA (*OlexA:CIPK26^TD^-YFP-HA*) or inactive CIPK26^KR^-YFP-HA (*OlexA:CIPK26^KR^-YFP-HA*) under the control of an estradiol-inducible promoter were generated. Transgenic *OlexA:CIPK26^TD^-YFP-HA, 35S:HA-KEG/keg-1*, and *OlexA:CIPK26^KR^-YFP-HA, 35S:HA-KEG/keg-1* seedlings were treated without or with 17-β-estradiol to induce expression of CIPK26^TD^-YFP-HA or CIPK26^KR^-YFP-HA, respectively. A cell-free degradation assay was then used to assess the effect of CIPK26 on KEG abundance. The level of HA-KEG was observed to decrease more rapidly over time when the active CIPK26^TD^-YFP-HA was induced, as compared to when the inactive kinase, CIPK26^KR^-YFP-HA, was induced (**Figure 2A**). Similarly, the level of HA-KEG was observed to decrease more rapidly over time when CIPK26^TD^-YFP-HA was induced, as compared to when CIPK26^TD^-YFP-HA was not induced (**Figure 2A** and **Supplementary Figure S3B**).

To further confirm these findings, the double-transgenic seedlings were used in a cycloheximide (CHX) chase assay to assess the stability of HA-KEG in the presence and absence of an active kinase. Seedlings were first treated with 17-β-estradiol to induce expression of either CIPK26^TD^-YFP-HA, or CIPK26^KR^-YFP-HA and then the media was supplemented with cycloheximide and plant tissue was collected at the indicated times. Samples from *OlexA:CIPK26^TD^-YFP-HA, 35S:HA-KEG/keg-1* seedlings revealed that the abundance of HA-KEG decreased considerably over time compared to samples taken from *OlexA:CIPK26^KR^-YFP-HA, 35S:HA-KEG/keg-1* seedlings (**Figure 2B**). The results from these assays suggest that the active CIPK26 promotes the degradation of KEG.

### Activation of CIPK26 Increases Stability of the Kinase

**FIGURE 3:**
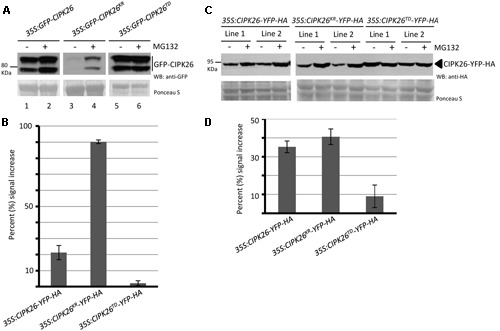
**A constitutively active version of CIPK26 is stable compared to the wild type and an inactive version. (A)** WB analysis showing the abundance of GFP-CIPK26, GFP-CIPK26^KR^, GFP-CIPK26^TD^ in transiently transformed *N. benthamiana* leaves treated with (+) or without (–) 50 μM MG132. Ponceau S staining was used to confirm loading. **(B)** Protein abundance of CIPK26-YFP-HA, CIPK26^KR^-YFP-HA, CIPK26^TD^-YFP-HA in two independent stable transgenic Arabidopsis plant lines treated (+) or without (–) 50 μM MG132. Ponceau S staining was used to confirm loading. **(C)** The graph illustrates the percent signal increase after MG132 treatment from the experiment shown in **(A)**. Signal intensities from each blot was determined using IMAGEJ software and shown as a percentage (%) increase of untreated (– MG132, control). Values are averages from two trials. The error bar represents ± standard error. **(D)** The graph illustrates the percent signal increase after MG132 treatment from the experiment shown in **(B)**. Values are averages from both independent lines across two trials. The error bar represents ± standard error.

Calcineurin B-like interacting protein kinase 26 is ubiquitinated *in planta* and degraded through the 26S proteasome (Lyzenga et al., 2013). Since an active CIPK26 promotes the degradation of KEG we sought to investigate how the kinase activity of CIPK26 influences its own stability. We assessed the stability of an inactive (GFP-CIPK26^KR^) and a constitutively active (GFP-CIPK26^TD^) version of CIPK26. Initially, we examined the accumulation of GFP-CIPK26, GFP-CIPK26^KR^, and GFP-CIPK26^TD^ in the presence and absence of proteasome inhibitor, MG132, using transient expression in tobacco. MG132 inhibits the 26S proteasome and causes accumulation of unstable proteins. By comparing the relative amounts of protein from an untreated sample to that of an MG132 treated sample, we can assess relative stability. We infiltrated Agrobacterium containing either *35S:GFP-CIPK26*, *35:GFP-CIPK26^KR^* or *35S:GFP-CIPK26^TD^* into a tobacco leaf on both sides of the mid-vein. One side of the mid-vein was treated with DMSO (control) and the other side with MG132. As shown in **Figures 3A,B**, the MG132 treated tissue accumulated more GFP-CIPK26 compared to the untreated sample (compare lanes 1 and 2). MG132-induced accumulation of GFP-CIPK26 is consistent with previous work showing that CIPK26 is unstable (Lyzenga et al., 2013). The two migrating forms corresponding to GFP-CIPK26 were also observed in a stable Arabidopsis *35S:GFP-CIPK26* transgenic line. Interestingly, GFP-CIPK26^KR^ is barely detected in the absence of proteasome inhibitors, while a strong increase in GFP-CIPK26^KR^ abundance is observed following treatment with MG132 (**Figure 3A**, compare lanes 3 and 4). Unlike GFP-CIPK26 and GFP-CIPK26^KR^, the abundance of GFP-CIPK26^TD^ did not show any substantial increase following treatment with the proteasome inhibitor (**Figure 3A**, compare lanes 5 and 6).

To investigate if this trend in protein stability would occur in Arabidopsis, two independent stable transgenic lines expressing C-terminal yellow fluorescence protein (YFP) and hemagglutinin (HA) tagged versions of CIPK26, CIPK26^KR^, or CIPK26^TD^ were generated. Unlike the GFP-CIPK26 fusion protein, CIPK26-YFP-HA did not migrate as two forms. However, similar to the results observed in the transient assays, MG132 treatment resulted in a marked accumulation of CIPK26^KR^-YFP-HA, while CIPK26^TD^-YFP-HA abundance was comparable in MG132 treated and untreated plant tissue (**Figures 3C,D**). These results suggest that the inactive kinase is very unstable and more rapidly degraded compared to wild type CIPK26. The results also suggest that when CIPK26 is active, it is relatively stable and not as efficiently turned over by the proteasome.

**FIGURE 4:**
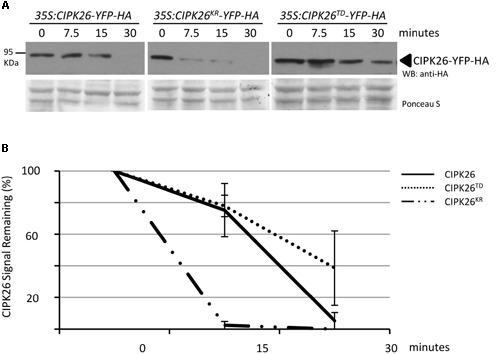
**Cell free degradation assay showing increased stability of an active CIPK26. (A)** The levels of CIPK26-YFP-HA at indicated time points were determined in total protein extracts from 4-day-old *35S:CIPK26-YFP-HA* (line 1), *35S:CIPK26^KR^-YFP-HA* (line 1), and *35S: CIPK26^TD^-YFP-HA* (line1) seedlings by WB with HA antibody. Blot is representative of two trials. Ponceau S staining was used to confirm equal loading. **(B)** Quantification of two trials using signal intensity at each time point determined by IMAGEJ. The error bar represents ± standard error.

To further compare the stability of CIPK26-YFP-HA, CIPK26^KR^-YFP-HA, and CIPK26^TD^-YFP-HA we used a cell-free degradation assay. Protein extracts prepared from 4-day-old *35S:CIPK26-YFP-HA, 35S:CIPK26^KR^-YFP-HA*, and *35S:CIPK26^TD^-YFP-HA* transgenic seedlings were used in the assay. Samples were collected at the time points indicated and the level of each protein was determined. Consistent with the above results, the abundance CIPK26^KR^-YFP-HA was found to be decrease more rapidly compared to CIPK26-YFP-HA, while CIPK26^TD^-YFP-HA was found to relatively consistent when compare to the other kinase variants (**Figures 4A,B** and **Supplementary Figure S3C**).

## Discussion

**FIGURE 5:**
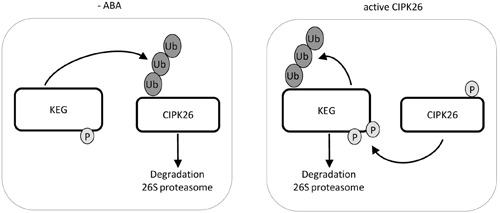
**Model of KEG and CIPK26 reciprocal regulation.** In the absence of ABA, KEG ubiquitinates CIPK26 and the modified kinase is degraded through the 26S proteasome. When CIPK26 is active, CIPK26 is stable and phosphorylates KEG which promotes the ubiquitination of KEG and subsequent degradation through the 26S proteasome.

The RING-type E3 ligase KEG plays a multifaceted role in ABA signaling as it regulates the abundance of ABA-related transcription factors ABI5, ABF1, and ABF3 along with an ABA-related kinase CIPK26 (Stone et al., 2006; Liu and Stone, 2010, 2013; Chen et al., 2013; Lyzenga et al., 2013). An important theme of KEG’s regulation is its phosphorylation dependent self-ubiquitination and degradation in the presence of ABA (Liu and Stone, 2010). We have previously shown that KEG contributes to the ubiquitin-mediated degradation of CIPK26 (Lyzenga et al., 2013). However, conditions that affect the stability of CIPK26 have not been investigated and upstream signaling events that phosphorylate KEG and promote KEG degradation have not been established. By using a constitutively active version of CIPK26, we show that the activation of the kinase increases stability and promotes the degradation of KEG. We demonstrated that CIPK26 can phosphorylate KEG *in vitro* and provide *in planta* evidence that an activated CIPK26 enhances KEG degradation. Based on these results we suggest a model wherein CIPK26 phosphorylates KEG to promote degradation of the E3 (**Figure 5**). The phosphorylation of KEG by CIPK26 may be in response to ABA or other abiotic stress and provide a link between the perception of ABA and KEG protein levels.

Cross-talk between phosphorylation and ubiquitination can take many different forms and can act on the kinase, E3 ligase and/or targets. Phosphorylation of the E3 ligase can influence activity through various mechanisms. For example, phosphorylation of a specific residue of the mammalian RING-type E3 ligases, Cbl, enhances E3 ligase activity by preventing the RING domain from adopting an auto-inhibited state and by promoting a conformational change that is favorable for ubiquitin transfer (Dou et al., 2012). Phosphorylation of the extensively studied mammalian RING-type E3 ligase, Mdm2, enhances self-ubiquitination as a form of self-regulation (Metzger et al., 2014). Phosphorylation of ubiquitination targets may impact specific modular binding domains thereby creating or destroying phosphodegrons. We generated at constitutively active version of CIPK26 by making a phosphomimic mutation in the activation loop of CIPK26 (CIPK26^TD^) (Gong et al., 2002a,b; Lyzenga et al., 2013). The phosphomimic mutation in the activation loop stabilizes substrate binding and an active conformation (Nolen et al., 2004). Constitutive activation or inactivation of a kinase is known to modulate degradation by the UPS (Lu and Hunter, 2009). Here, the constitutively active version of CIPK26 is stable, while the kinase dead CIPK26 is rapidly degraded. There may be a two-fold reason as to why the active CIPK26 is stable. First, the self-phosphorylation activity of CIPK26 may disrupt a degron sequence within CIPK26 thereby preventing ubiquitination. Secondly, CIPK26 may phosphorylate KEG to affect E3 activity and/or substrate specificity. Results from this and previous reports (Lyzenga et al., 2013) provide support for the latter, which suggests that an active and stabilized CIPK26 promotes KEG self-regulation.

Previous work has shown that a general kinase inhibitor prevents the ABA-induced degradation of KEG, and that a phosphorylated KEG has increased self-ubiquitination activity compared to the unphosphorylated form (Liu and Stone, 2010). KEG also contains a kinase domain. KEG harboring an inactivated kinase is more stable and less ubiquitinated in the presence of ABA than wild type KEG. This evidence suggests that phosphorylation plays an important role in negatively regulating KEG activity through self-ubiquitination. In this report, we show that CIPK26 can phosphorylate KEG *in vitro*. Using modified cell-free degradation and cycloheximide chase assays to monitor protein turnover, we show that the activated CIPK26 regulates KEG stability, promoting degradation of the E3. ABA and/or other signals may activate CIPK26, which increases its stability and in turn phosphorylates KEG to negatively regulate the E3 ligase by promoting KEG degradation (**Figure 5**). Interestingly, CIPK26 also interacts with SRK2D which functions as a key positive regulator of ABA signaling (Fujita et al., 2009; Mogami et al., 2015). However, a *cipk26/3/9/23* quadruple mutant did not display an ABA related phenotype during vegetative development (Mogami et al., 2015). CIPK26 appears to share functions with the homologs *CIPK3*, *CIPK9* and *CIPK23* as the quadruple mutant displays impaired growth phenotypes (Mogami et al., 2015). The interplay between CIPK26 and ABA signaling components may be very complex and developmentally specific.

Our study shows that a constitutively active CIPK26 is more stable than the wild type and an inactive version. According to our model, in the absence of a stimulus wild type CIPK26 should be inactive and therefore turned over in a manner similar to the kinase dead version. However, the kinase dead CIPK26 was turned over more rapidly compared to the wild type kinase. The clade A PP2Cs, including ABI1/2, probably counteract the activity of CIPK26 similar to how the phosphatases inactivate other SnRK family kinases during early ABA receptor-binding events (Lee et al., 2007; Lan et al., 2011; Lyzenga et al., 2013). Overexpression of wild type CIPK26 may lead to limited escape from ABI1/2-mediated dephosphorylation and subsequent activation of the kinase in the absence of a stimulus. Interestingly, basal activity has been reported for some members of the CIPK family and this basal level activity may account for why wild type CIPK26 is not as highly unstable as the inactivated version of the kinase (Guo et al., 2001; Zhou et al., 2014). An intriguing thought is the possibility that the basal activity of the CIPKs may represent a pool of stable kinases that may aid in initiating KEG self-regulation.

Many activated kinases are degraded through the proteasome as a form of down regulation (Hunter, 2007; Lu and Hunter, 2009). For example, SnRK1, the plant ortholog of mammalian AMPK and SNF from yeast and a member of the SNF1-related kinase super family (along with CIPK/SnRK3 and SnRK2s), is degraded through the proteasome after activation (Coello et al., 2012; Crozet et al., 2016). Attenuation of CIPK26 function may be achieved using an alternate E3 ligase. This may account for the decrease in the levels of the activated kinase observed following induction of expression in *in planta* assays. Alternatively, downregulation of CIPK26 may be achieved through selective dephosphorylation. In addition, activation of CIPK kinases incorporates binding of Ca^2+^sensing CBL proteins to the self-inhibitory NAF motif, activation loop phosphorylation by upstream kinases and phosphorylation of the CBL by the CIPK (Chaves-Sanjuan et al., 2014; Sanyal et al., 2015). Developmental phases or abiotic stress may promote CIPK26 activation, self-phosphorylation and subsequent stabilization. These multiple levels of regulation may function to fine tune CIPK26 kinase activity.

## Author Contributions

WJL, VS, and HL carried out the experiments and participated in experimental design along with SLS. WJL and SLS wrote the manuscript.

## Conflict of Interest Statement

The authors declare that the research was conducted in the absence of any commercial or financial relationships that could be construed as a potential conflict of interest.

## Footnotes

1 http://www.qiagenbioinformatics.com

## Supplementary Material

FIGURE S1 **Keep on Going (KEG) phosphorylation sites. (A)** Schematic representation of KEG protein showing phosphorylation sites retrieved from Plant Protein Phosphorylation Database (P3DB) and the Arabidopsis Protein Phosphorylation Site Database (PhosPhAt 4.0). **(B)** Segments of an alignment using KEG amino acid sequences from 10 plant species. Phosphorylation sites from **(A)** are indicated with arrows. Numbers indicate amino acid position in *Arabidopsis thaliana* KEG.

FIGURE S2 **Amino acid sequence alignment of KEG from different plant species**. The complete amino acid sequence was used in the alignment.

FIGURE S3 **(A)** Modified cell free degradation assay in which purified Flag-His-CIPK26N^TD^ or Flag-His-CIPK26N^KR^ was added to protein extracts prepared from 6-day-old *35S:HA-KEG* seedlings. HA-KEG protein levels were determined by western blotting (WB) using HA antibodies at the indicated time points. **(B)** The constitutively active CIPK26 promotes KEG degradation *in planta*. Cell-free degradation assay using protein extracts from 5-day-old *OLexA:CIPK26^TD^-YFP-HA, 35S:HA-KEG/keg-1* (line 2) seedlings induced to express CIPK26^TD^-YFP-HA with 20 μM 17-ββ-estradiol. HA-KEG protein abundance was determined by western blotting (WB) using HA antibodies at the indicated time points. Ponceau S staining was used to confirm loading. **(C)** Cell free degradation assay showing increased stability of an active CIPK26. The levels of CIPK26-YFP-HA at indicated time points were determined in total protein extracts from four4-day-old *35S:CIPK26-YFP-HA* (line 1), *35S:CIPK26^KR^-YFP-HA* (line 1), and *35S: CIPK26^TD^-YFP-HA* (line1) seedlings by western blotting (WB) with HA antibody.

FILE S1 **Amino acid sequence alignment of KEG from different plant species (FASTA)**.
