# Supplementary figures and images for: The Kinase Activity of Calcineurin B-like Interacting Protein Kinase 26 (CIPK26) Influences Its Own Stability and that of the ABA-regulated Ubiquitin Ligase, Keep on Going (KEG)
Source: Front Plant Sci. 2017 Apr 10;8:502. doi: 10.3389/fpls.2017.00502 (PMC5385374; doi:10.3389/fpls.2017.00502)

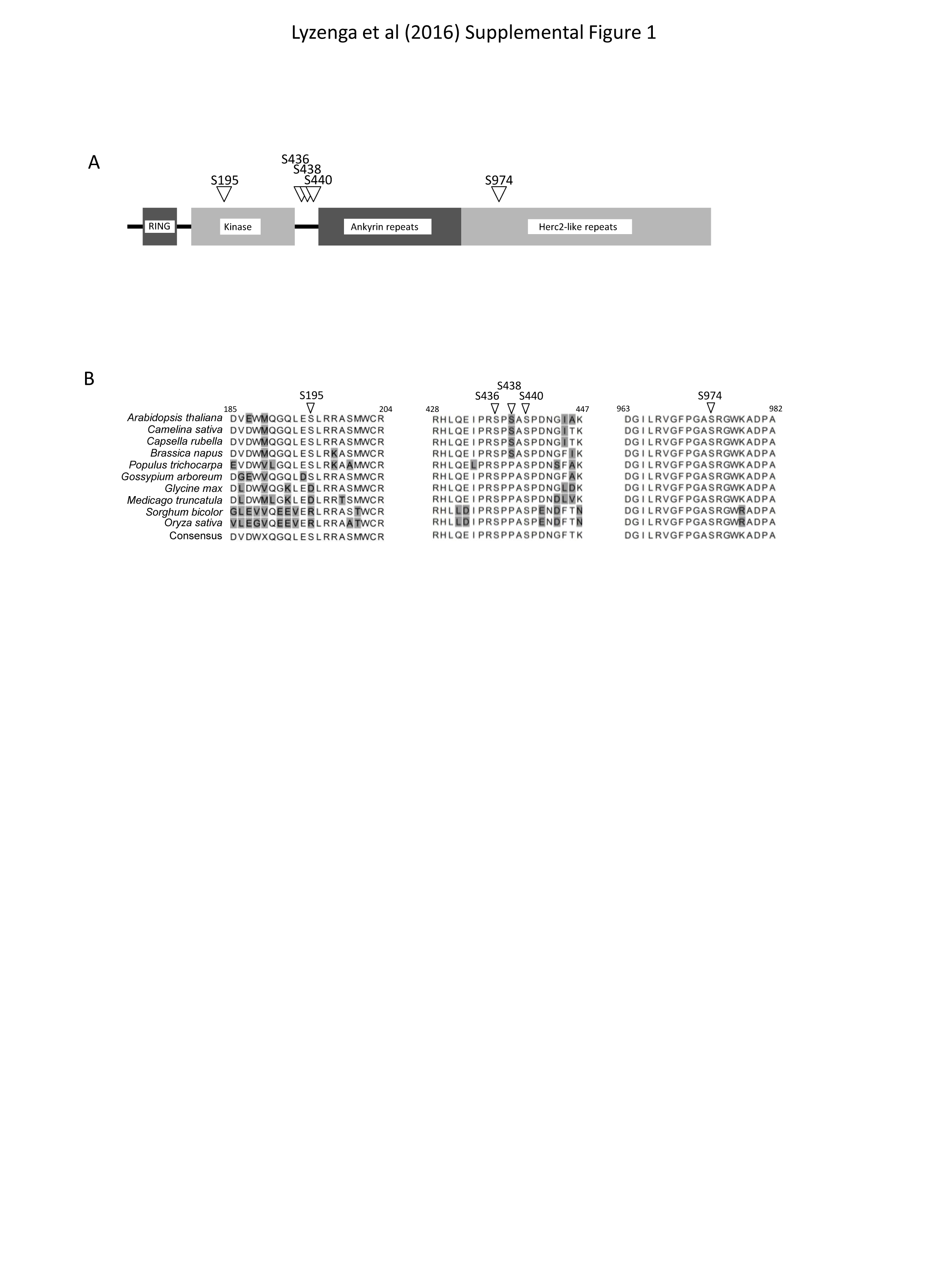

Supplement: FIGURE S1 — Keep on Going (KEG) phosphorylation sites. (A) Schematic representation of KEG protein showing phosphorylation sites retrieved from Plant Protein Phosphorylation Database (P3DB) and the Arabidopsis Protein Phosphorylation Site Database (PhosPhAt 4.0). (B) Segments of an alignment using KEG amino acid sequences from 10 plant species. Phosphorylation sites from (A) are indicated with arrows. Numbers indicate amino acid position in Arabidopsis thaliana KEG. [file Image_1.TIF]

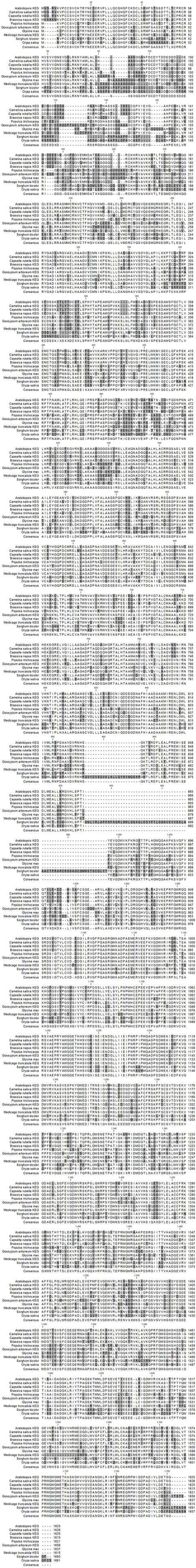

Supplement: FIGURE S2 — Amino acid sequence alignment of KEG from different plant species. The complete amino acid sequence was used in the alignment. [file Image_2.tif]

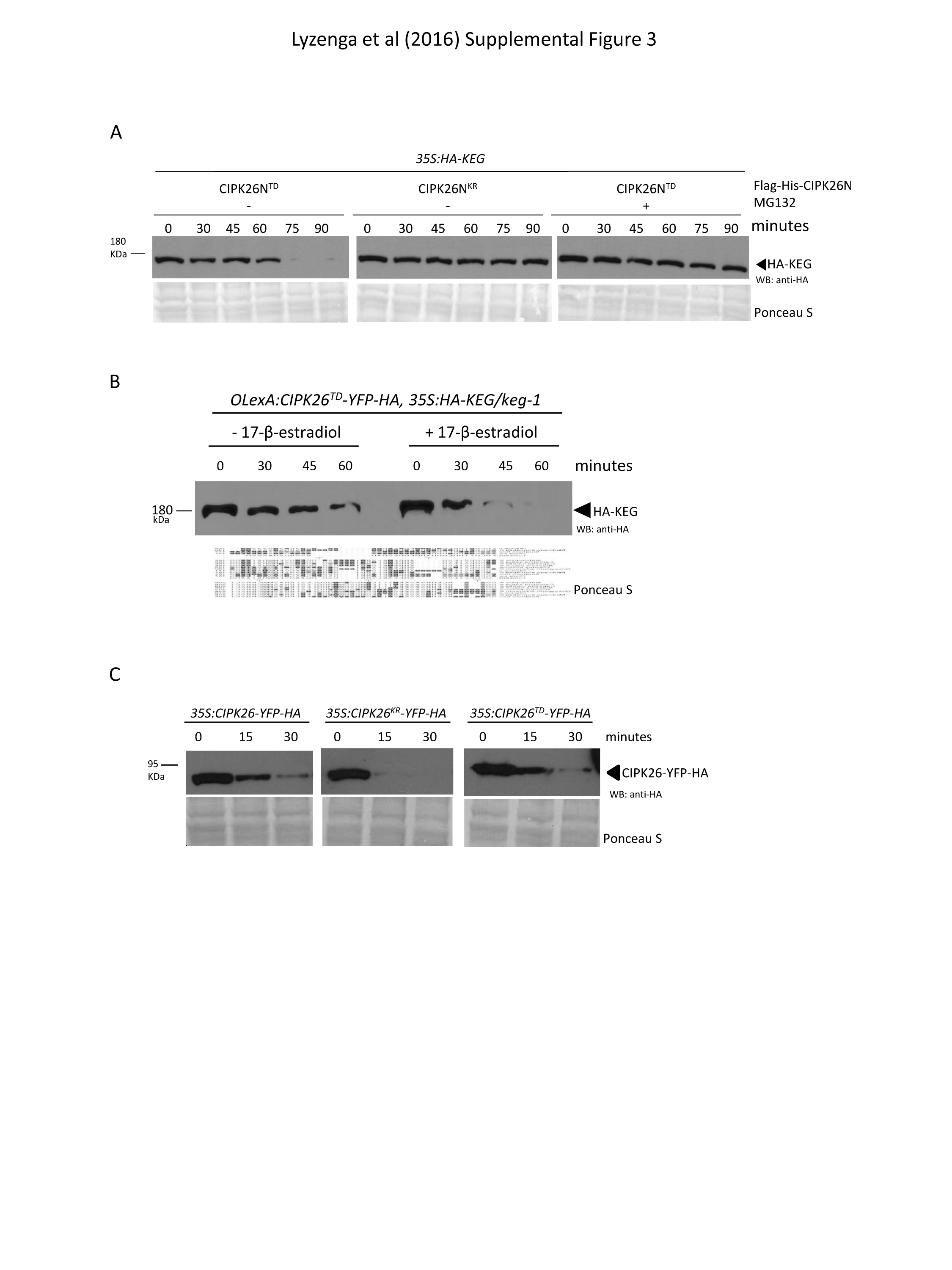

Supplement: FIGURE S3 — (A) Modified cell free degradation assay in which purified Flag-His-CIPK26NTD or Flag-His-CIPK26NKR was added to protein extracts prepared from 6-day-old 35S:HA-KEG seedlings. HA-KEG protein levels were determined by western blotting (WB) using HA antibodies at the indicated time points. (B) The constitutively active CIPK26 promotes KEG degradation in planta. Cell-free degradation assay using protein extracts from 5-day-old OLexA:CIPK26TD-YFP-HA, 35S:HA-KEG/keg-1 (line 2) seedlings induced to express CIPK26TD-YFP-HA with 20 μM 17-ββ-estradiol. HA-KEG protein abundance was determined by western blotting (WB) using HA antibodies at the indicated time points. Ponceau S staining was used to confirm loading. (C) Cell free degradation assay showing increased stability of an active CIPK26. The levels of CIPK26-YFP-HA at indicated time points were determined in total protein extracts from four4-day-old 35S:CIPK26-YFP-HA (line 1), 35S:CIPK26KR-YFP-HA (line 1), and 35S: CIPK26TD-YFP-HA (line1) seedlings by western blotting (WB) with HA antibody. [file Image_3.TIF]
