# Supplementary material for: The Kinase Activity of Calcineurin B-like Interacting Protein Kinase 26 (CIPK26) Influences Its Own Stability and that of the ABA-regulated Ubiquitin Ligase, Keep on Going (KEG)
Source: Front Plant Sci. 2017 Apr 10;8:502. doi: 10.3389/fpls.2017.00502 (PMC5385374; doi:10.3389/fpls.2017.00502)
Supplement: FILE S1 — Amino acid sequence alignment of KEG from different plant species (FASTA). [file Data_Sheet_1.DOCX]

>Arabidopsis_KEG

MVG-RVKVPCCSVCHTRYNEDERVPLLLQCGHGFCKDCLSKMFSTSSDTT

LTCPRCRHVSVVGNSVQGLRKNYAMLALIH-----------AASGGANFD

CDYTDDEDDD--DEEDGSDEDGA---------RAARGFHASSS--INSLC

GPVIEVG-----AHPEMKLVRQIGEESSS-GGFGGVEMWDATVAGGGG--

----RCKHRVAVKKMTLTEDMDVEWMQGQLESLRRASMWCRNVCTFHGVV

KMD-GSLCLLMDRCFGSVQSEMQRNEGRLTLEQILRYGADVARGVAELHA

AGVICMNIKPSNLLLDASGNAVVSDYGLAPILKKPTCQKTRPEFDSSKVT

LYTDCVTLSPHYTAPEAWGPVKK---LFWEDASGVSPESDAWSFGCTLVE

MCTGSTPWDGLSREEIFQAVVKARKVPPQYERIVGVGIPRELWKMIGECL

QFKPSKRPTFNAMLATFLRHLQEIPRSPSASPDNGIAKICEVNIVQAPRA

TN-IGVFQDNPNNLHRVVLEGDFEGVRNILAKAAAGGGGSSVRSLLEAQN

ADGQSALHLACRRGSAELVEAILEYGEANVDIVDKDGDPPLVFALAAGSP

QCVHVLIKKGANVRSRLREGSGPSVAHVCSYHGQPDCMRELLVAGADPNA

VDDEGETVLHRAVAKKYTDCAIVILENGGSRSMTVSNAKCLTPLHMCVAT

WNVAVIKRWVEVSSPEEISQAINIPSPVGTALCMAASIRKDHEKEGRELV

QILLAAGADPTAQDAQHGRTALHTAAMANNVELVRVILDAGVNANIRNVH

NTIPLHMALARGANSCVSLLLESGSDCNIQDDEGDNAFHIAADAAKMIRE

NLDWLIVMLRSPDAAVDVRNHS----------------------GKTVRD

FLEALPREWISEDLMEALLKRGVHLSPTI---------------------

---------------------------------------------YEVGD

WVKFKRGITTPLHGWQGAKPKSVGFVQTILEKED--MIIAFCSGE--ARV

LANEVVKLIPLDRGQHVRLRADVKEPRFGWRGQSRDSVGTVLCVD-EDGI

LRVGFPGASRGWKADPAEMERVEEFKVGDWVRIRQNLTSAKHGFGSVVPG

SMGIVYCVRPDSSLLVELSYLPNPWHCEPEEVEPVAPFRIGDRVCVKRSV

AEPRYAWGGETHHSVGKISEIENDGLLIIEIPNRPIPWQADPSDMEKIDD

FKVGDWVRVKASVSSPKYGWEDITRNSIGVMHSLDEDGDVGIAFCFRSKP

FSCSVTDVEKVTPFHVGQEIHMTPSITQPRLGWSNETPATIGKVMRIDMD

GTLSAQVTGRQTLWRVSPGDAELLSGFEVGDWVRSKPSLGNRPSYDWSNV

GRESIAVVHSIQETGYLELACCFRKGRWSTHYTDLEKIPALKVGQFVHFQ

KGITEPRWGWRAAKPDSRGIITTVHADGEVRVAFFGLPGLWRGDPADLEV

EPMFEVGEWVRLREGVSCWKSVGPGSVGVVHGVGYEGDEWDGTTSVSFCG

EQERWAGPTSHLEKAKKLVVGQKTRVKLAVKQPRFGWSGHSHGSVGTISA

IDADGKLRIYTPAGSKTWMLDPSEVETIEEEE-LKIGDWVRVKASITTPT

YQWGEVNPSSTGVVHRMEDGDLCVSFCFLDRLWLCKAGELERIRPFRIGD

RVKIKDGLVTPRWGWGMETHASKGHVVGVDANGKLRIKFLWREGRPWIGD

PADIVLDETSG----------

>Camelina_sativa_KEG

MVG-RVKVPCCSVCHTRYNEDERVPLLLQCGHGFCKDCLSKMFSTSSDTT

LTCPRCRHVSVVGNSVQGLRKNYAMLALIH-----------AASGGANFD

CDYTDDEDED--DEDDGSDEDRA---------RSARGFLASSS--INSSC

GPVIEVG-----AHPEMKLGRQIGEESSG-GGFGGVEMWDATVAGGGG--

----RCKHRVAVKKMTLTEDMDVDWMQGQLESLRRASMWCRNVCTFHGVV

KME-GSLCLLMDRCYGSVQSEMQRNEGRLTLEQILRYGADVARGVAELHA

AGVICMNIKPSNLLLDANGNAVVSDYGLAPILKKPTCQKTRPEYDSSKLT

LYTDCVTLSPHYTAPEAWGPVKK---LFWEDASGVSPESDAWSFGCTLVE

MCTGSTPWDGLSREEIFQAVVKARKVPPQYERIVGVGIPRELWKMIGECL

QFKPSKRPTFNAMLATFLRHLQEIPRSPSASPDNGITKICEVNIVEATRP

TNNIGVFQDNPNNLHRVVLEGDFEGVRNILAKAAAGGGGSSVRSLLEAQN

ADGQSALHLACRRGSAELVEAILEYGEASVDIVDKDGDPPLVFALAAGSP

QCVHVLIKKGANVRSRLREGSGPSVAHVCSYHGQPDCMRELMVAGADPNA

VDDEGETVLHRAIAKKYTDCAIVILENGGSRSMAVSNAKCLTPLHMCVAT

WNVAVIKRWVEVSTPEEISQAINIPSPVGTALCMAASIKKDHEKEGRELV

QILLAAGADPTAQDSQHGRTALHTAAMANNVELVRVILDAGVNANIRNVH

NTIPLHMALARGANSCVSLLLESGSDCNIQDDEGDNAFHIAADAAKMIRE

NLDWLIVMLRSPDAAVDVRNHS----------------------GKTVRD

FLEALPREWISEDLMEALLKRGVHLSPTI---------------------

---------------------------------------------YEVGD

WVKFKRGITTPLHGWQGAKPKSVGFVQTILEKED--MIVAFCSGE--ARV

LANEVVKLIPLDRGQHVRLRADVKEPRFGWRGQSRDSVGTVLCVD-EDGI

LRVGFPGASRGWKADPAEMERVEEFKVGDWVRIRQNLTSAKHGFGSVVPG

SMGIVYCVRPDSSLLVELSYLPNPWHCEPEEVEPVAPFRIGDRVCVKRSV

AEPRYAWGGETHHSVGKISEIENDGLLIIEIPNRPIPWQADPSDMEKIDD

FKVGDWVRVKASVSSPKYGWEDITRNSIGVMHSLDEDGDVGIAFCFRSKP

FSCSVTDVEKVVPFHVGEEIHMTPSITQPRLGWSNETPATIGKIMRIDMD

GTLSAQVTGRQTLWRVSPGDAELLSGFEVGDWVRSKPSLGNRPSYDWFSV

GRESIAVVHSIQETGYLELACCFRKGRWSTHYTDLEKIPALKVGQFVHFQ

KGLTEPRWGWRGAKPDSRGIITTVHADAEVRVAFFGLPGLWRGDPADLEV

EPMFEVGKWVKLREGAPSWKSAGPGSVGVVHGVGYEGDEWDGTTSVSFCG

EQERWAGSSSHLEKAKKLVVGQKTRVKLAVKQPRFGWSGHSHGSIGTIAA

IDADGKLRIYTPAGSKTWMLDPSEVETIEEEE-LRIGDWVRVKASITTPT

YQWGEVNPSSIGVVHRMEDGDLWVSFCFLDRLWLCKAGELERIRPFRIGD

RVKIKDGLVTPRWGWGMETHASKGHVVGVDANGKLRIKFLWREGRPWIGD

PADIVLDETSG----------

>Capsella_rubella_KEG

MVG-RVKVPCCSVCHTRYNEDERVPLLLQCGHGFCKDCLSKMFSTSSDTT

LTCPRCRHVSVVGNSVQGLRKNYAMLALIH-----------AASGGPNFD

CDYTDDDDDDEDDEDDSSDEDRA---------RSPRGFHASTS--INSSC

GPVIEVG-----AHPEMKLVRQIGEES-G-AGFGGVQMWDATVAGGGG--

----RCKHRVAVKKITLTEDMDVDWMQGQLESLRRASMWCRNVCTFHGVV

KMK-ASLCLLMDRCYGSVQSEMQRNEGRLTLEQILRYGADVARGVAELHA

AGVICMNIKPSNLLLDASGNAVVSDYGLAPILKKPTCQKTRPEYDSSKLT

LYTDCVTLSPHYTAPEAWGPVKK---LFWEDASGVSPESDAWSFGCTLVE

MCTGSTPWDGLSREEIFQAVVKARKVPPQYERIVGVGIPRELWKMIGECL

QFKPSKRPTFNAMLATFLRHLQEIPRSPSASPDNGITKICEVNIVEATRA

TN-IGVFQDNPNTLHRVILEGDCERVRNILAKAAAGGGGSSVRSLLEAQN

ADGQSALHLACRRGSAELVEVILEYGEANVDIVDKDGDPPLVFALAAGSP

QCVHVLIKKGANVRSRLREGSGPSVAHVCSYHGQPDCMRELLVAGADPNA

VDDEGETVLHRAVSKKYTDCAVVVLENGGSRSMAVSNAKCLTPLHMCVAT

WNVAVIKRWVEVSSPEEISQAINIPSSVGTALCMAASLKKDHEK-GRELV

QILLAAGADPTAQDSQHGRTALHTAAMANNVELVRVILDAGVNANIRNVH

NTIPLHMALARGANSCVSLLLESGSDCNIQDDEGDNAFHIAADAAKMIRE

NLDWLIVMLRSPDAAVGVRNHS----------------------GKTVRD

FIEALPREWISEDLMEALLKRGVHLSPTI---------------------

---------------------------------------------YEVGD

WVKFKRGITTPLHGWQGAKPKSVGFVQTILEKED--MIVAFCSGE--ARV

LASEVIKLIPLDRGQHVRLRADVKEPRFGWRGQSRDSVGTVLCVD-EDGI

LRVGFPGASRGWKADPAEMERVEEFKVGDWVRIRQNLTSAKHGFGSVVPG

SMGIVYCVRPDSSLLVELSYLPNPWHCEPEEVEPVAPFRIGDRVCVKRSV

AEPRYAWGGETHHSVGRISEIENDGLLIIEIPNRPIPWQADPSDMEKIDN

FKVGDWVRVKASVSSPKYGWEDITRNSIGVMHSLDEDGDVGIAFCFRSKP

FSCSVTDVEKVVPFHVGQEIHMIPSITQPRLGWSNETPATIGKIMRIDMD

GTLSAQVTGRQILWRVSPGDAELLSGFEVGDWVRSKPSLGNRPSYDWFSV

GRESIAVVHSIQEAGYLELACCFRKGRWSTHYTDLEKIPALKVGQFVHFQ

KGLTEPRWGWRGAKPDSRGIITTVHADGEIRVAFFGLPGLWRGDPADLEV

EPMFEVGEWVRLREGVPSWKTVGPGSVGVVHGVGYEGDEWDGTTSVSFCG

EQERWAGSSSHLEKAKKLVVGQKTRVKLAVKQPRFGWSGHSHGSIGTIAA

IDADGKLRIYTPAGSKTWMLDPSEVETIEEEE-LRIGDWVRVKASITTPT

YQWGEVNPSSIGVVHRMEDGDLWVSFCFLDRLWLCKAAELERVRPFRMGD

PVKIKDGLVTPRWGWGMETYASKGHVVGVDANGKLRIKFLWREGRPWIGD

PADIVLDETSG----------

>Brassica_napus_KEG

MVAAKMKVPCCSVCHTRYNEDERVPLLLQCGHGFCKDCLSKMFSSSSDTS

LACPRCRHVSVVGNSVQGLRKNFAMLALV---------------GGGNFD

CDYTDS-DEDEDDEED--DRYAA---------SSSRGGDKSSS------C

GPVIEVG-----AHPEMKLVKRIGEDGSG-GG--GVEMWDATVAGGGG--

----RCKHRVAVKKMCLTEDMDVDWMQGQLESLRKASMWCRNVCTFHGVV

KMD-GSMCLLMDRCFGSVQSEMERNEGRLTLEQILRYGADVARGVAELHA

AGVICMNIKPSNLLLDASGNAVVSDYGLAPILKKPICQKTRPEFDSSKIT

PYTDCVTLSPHYTAPEAWGPVKK---LFWEDASGVSPESDAWSFGCTLVE

MCTGSTPWDGLSREEIFQAVVKARKVPPQYERIVGVGIPRELWKMIGECL

QFKPSKRPTFNAMLATFLRHLQEIPRSPSASPDNGFIKVCRVNVVEETRS

TN-MGVLPDNPINLHRVVLEGDSEGVRNILAKAATGSGGSSVRYLLEAQN

ADGQSALHLACRRGSVELVEAILEYGEANVDIVDKDGDPPLVFALAAGSP

QCVHVLIKKGANVRSRLREGSGPSVAHVCSYHGQPDCMRELLVAGADPNA

VDDEGETVLHRAVTKKYTDCAIVILENGGSRSMAVSNGKGLTPLHMCVST

WNVAVIKRWVEVSSPEEISQAIDIPSPVGTALCMAAAIRKDHEKEGRELV

QILLAAGADPTAQDAQHGRTALHTAAMANNVELVRVILDAGVNANIRNVH

NTIPLHMALARGANACVSLLLESGSDCNIQDDEGDNAFHIAADAAKMIRE

NLDWLIVMLRRPDAAVDVRNHS----------------------GKTVRD

FLEALPREWISEDLMEALLKRGVHLSPTI---------------------

---------------------------------------------YEIGD

WVKFKRGITTPLHGWQGAKPKSVGFVQTILEKED--MIVAFCSGE--ARV

LASEVVKLIPLDRGQHVRLRKDVKEPRFGWRGQSRDSVGTVLCVD-EDGI

LRVGFPGASRGWKADPAEMERVEEFKVGDWVRIRQNLTSAKHGFGSVVPG

SMGIVYCVRPDSSLLVELSYLPNPWHCEPEEVEPATPFRIGDRVCVKRSV

AEPRYAWGGETHHSVGKISEIENDGLLIIEIPNRPIPWQADPSDMEKIDD

FKVGDWVRVKASVSSPKYGWEDITRNSIGVMHSLDEDGDVGIAFCFRSKP

FSCSVTDVEKVVPFHVGQEIHMIPSIAQPRLGWSNETPATIGKIMRIDMD

GTLSAQVTGRQTLWKVSPGDAEMLSGFEVGDWVRSKPSLGSRPSYDWFSV

GRDSIAVVHSIQEAGYLELACCFRKGRWSTHYTDLEKIPSLKAGQFVRFQ

KGLTEPRWGWRGAKPDTRGIITTVHADGEVRVAFFGLPGLWKGDPADLEV

ERMFEVGEWVRLKEGVPSWKSIGPGSVGVVHGVGYEGDEWDGTISVSFCG

EQERWTGSFTHLEKAKKLVVGQKTRVKLAVKQPRFGWSGHSHGSVGTIAA

IDADGKLRIYTPAGSKTWMLDPSEVETIEEEE-LKIGDWVRVKPSITTPT

YQWGEVNPSSIGVVHRMEDGDLWVSFCFLDRLWLCKAVEMERIRPFGIGD

KVKIKNGLVTPRWGWGMETHASKGHVVGVDANGKLRIKFLWREGRPWIGD

PADIVLDEPSG----------

>Populus_trichocarpa

M-----KVPCCSVCQTRYDEEERVPLLLQCGHGFCKDCLSRMFSASTDTT

LVCPRCRHVSVVGNSVTALKKNFAVLALLHS---SS-----SSSAAANFD

CDYTDDE-GDGDEEDFEEE-------------RCSRGSHASSSGA----C

GPVIDVG-----AHPEVKLVKKI---GEGRS-KSGMETWTAVIGG--GGV

HGKKVCRHRVAVKKVEIGEEMEVDWVLGQLESLRKAAMWCRNVCTFHGVV

KMD-GCLGIVTDRCYGSVESEMQRNEGRLTLEQILRYGADIARGVAELHA

AGVVCMNIKPSNLLLDSSGRAVVSDYGLAAILKKPACRKARSECDSAKIH

SCMDCTMLSPNYTAPEAWEPVKKSLNLFWDDAIGISVESDAWSFGCALVE

MCTGSIPWAVLSADEIYRAVVKGRKLPPQYASVVGVGMPRELWKMIGECL

QFKASKRPAFSAMLAIFLRHLQELPRSPPASPDNSFAKYPRSYVKEPP-L

ASDLEVFQDNPGHLHRFVSEGDVSGVRELLAKVASRNDNFPISMLLEAQN

ADGQTALHLACRRGSSELVRAILEYREADVDVLDKDGDPPLVFALAAGSP

ECVRALIERGANVRSRLREGFGPSVAHVCAYHGQPDCMRELLLAGADPNA

IDDEGESVLHRAVSKKYTDCALVILENGGCGSMAVPNSKNLTPLHLCVAT

WNVAVVRRWVEVASPEEIADAIDIPSPVGTALCMAAAAKKDHETEGRELV

RILLFAGADPTAQDAQHGRTALHTAAMANDVELVKIILDAGVDVNIRNVQ

NTIPLHVALARGAKSCVGLLLSAGANCNMQDDEGDNAFHIAAETAKMIRE

NLEWLILMLRNSNAAVEVRNHS----------------------GKTLRD

FLEALPREWISEDLMEALVNRGVHLSPTI---------------------

---------------------------------------------FEVGD

WVKFKRSVTTPTHGWQGAKHKSVGFVQTVVDKDN--LIVSFCSGE--ARV

LANEVLKVIPLDRGQHVQLKQDVKEPRFGWRGQSRDSIGTVLCVD-DDGI

LRVGFPGASRGWKADPAEMERVEEFKVGDWVRIRPTLTTAKHGLGSVTPG

SIGIVYCIRPDNSLLLELSYLPNPWHCEPEEVEPVAPFKIGDRVCVKRSV

AEPRYAWGGETHHSVGRISEIENDGLLIIEIPNRPIPWQADPSDMEKVED

FKVGDWVRVKASVSSPKYGWEDITRNSIGVIHSLEEDGDMGVAFCFRSKP

FCCSVTDVEKVPPFEMGQEIHVLSSVTQPRLGWSNESPATVGKIVRIDMD

GALNVRVTGRHSLWKVSPGDAERLSGFEVGDWVRSKPSLGTRPSYDWNSI

GKESLAVVHSIQETGYLELACCFRKGRWIAHHTDIEKVPCFKVGQHVRFR

TGLSEPRWGWRGAQPDSRGIITSVHADGEVRIAFFDLPGLWRGDPADLEV

EHIFEVGEWVKLRGDVSNWKSVGPGSVGVVQGIGYDGDEWDGSIYVGFCG

EQERWAGPTSHLERVERLMVGQKVRVKLSVKQPRFGWSGHSHGSVGTIAA

IDADGKLRIYTPVGSKTWMLDPSEVELVEDEE-LHIGDWVKVRASISTPT

HQWGEVNHSSTGVVHRMENGDLWVSFCFLEKLWLCKALEMERIRPFKVGD

KVKIREGLVTPRWGWGMETHASKGQVVGVDANGKLRIKFHWREGRPWIGD

PADIVLDES------------

>Gossypium_arboreum_KEG

M-----KVPCCSVCQTRYNEEERVPLLLQCGHGFCKECLSRMFSASSDTS

LPCPRCRHVSLVGNSVLALKKNYGILALLNSNPNSA-----GSNSRNDFD

CDYTDDE-GDDDDEGREDDDENGDFFHELTGGRINRGSHASSSGGAAAGC

GPVIELT-----AHPEVKLVRKIEGKGEGKGGRAGVETWAAVISGAYGGG

GGRR-CKHKVAVKKVGAMEGMDGEWVQGQLDSLRRASMWCRNVCTFHGVI

KLEESSLGIVMDRCHGSIQSAMFNNEGRLTLEQVLRYGADIARGVAELHA

AGVVCMNIKPSNLLLDANGHAVVSDYGLASILKNPACRKARAECDSSKIH

SCMDCTMLSPHYTAPEAWEPVKKSLNLFWEDAIGISVESDAWSFGCTLVE

MCTGSIPWAGLSADEIYRAVVKSRKLPPQYASVVGVGLPRELWKMIGDCL

QFKPSKRPTFNAMLAIFLRHLQEIPRSPPASPDNGFAKFPGSNVVEPP-A

VADLEVVPDNPNLLHRLISEGDVSGVRDFLANASSGNSGTSISSLLEAEN

ADGQTALHLACRRGSAELVEAILEYAEANVDVLDKDGDPPLVFALAAGSP

ECVLALIRRGSDVQSRLREGFGPSVAHVCAYYGQPDCMRELLLAGADPNA

VDDEGESVLHRAVAKKYTDCALVILENGGCASMAVLNSKNLTPLHLCVAT

WNVAVVKRWVEVASPEEIAEAIDIPSPVGTALCMAAALKKDHEIEGRELV

RLLLAAGADPTAQDGQHGRTALHTAAMANDVELVKIILDAGVDVNIRNVH

NTTPLHVALARGATSCVGLLLSAGADCNLQGDEGDNAFHIAADTAKMIRE

NLEWLIVMLRNPDAAVEVRNHS----------------------GKTLRD

FLETLPREWISEDLMEALMNRGVHLSPTL---------------------

---------------------------------------------FEVGD

WVKFRRRITTPTYGWQGARHKSVGFVQNVVDRDN--LIVSFCSGDGNARV

LVNEVVKVIPLDRGQHVKLRADVKEPRFGWRGQSRDSIGTVLCVD-DDGI

LRVGFPGASRGWKADPAEMERVEEFKVGDWVRIRPTLTTAKHGLGSVTPG

SIGIVYCIRPDSSLLLDLSYLPNPWHCEPEEVEPVSPFRTGDRVCVKRSV

AEPRYAWGGETHHSVGRISEIETDGLLIIEIPNRPIPWQADPSDMEKLED

FKVGDWVRVKASVPSPKYGWEDITRNSIGIIHSLEDDGDIGIAFCFRSKP

FCCSVTDVEKVPPFEVGQEVHVMPSVSQPRLGWSNETPATVGKIVRIDMD

GALNVRVSGRHSLWKLSPGDAERLSGFEVGDWVRSKPSLGTRPSYDWNTI

GKENLAVVHSIQDTGYLELACCFRKGKWSTHFSDVEKVPSYKVGQHVRFR

AGLVEPRWGWRGTQSDSRGIITSVHADGEVRVAFFALPGMWRGDPADFDI

EPMFGVGEWVQLRENASCWKSIGPGSVGVVQGIGYEGDEWDGSTLVAFCG

EQERWMGPTSHLEKVDRLVIGQKVRVKLSVKQPRFGWSGHSHTSVGTIAA

IDADGKLRIYTPVGSKTWMLDPSEVELIEEQE-LCIGDWVRVRPSISVPT

HHWGEVTHSSIGVVHRMENGDLWVAFCFTERLWLCKACEMERVRPFKVGD

KVRIREGLVTPRWGWGMETHASKGQVVGVDANGKLRIKFQWREGRPWIGD

PADIVLDDSFGITSTS-----

>Glycine_max

M-----KIPCCSVCQTRYNEEERVPLLLQCGHGFCRECLSRMFSASSDAT

LACPRCRHVSTVGNSVQALRKNYAVLALL----NSAAAANGGGGGRSSFD

CDCTDDEDGDGGGEDEEEDDE------KR--RRNSRESQASSSGG---GC

APVIELGGGGGGAHNDLKLVQRI---GEGR--RAGVEMWMAVISGG-GGE

VGRQRCRHNVAVKKVAVAEGMDLDWVQGKLEDLRRASMWCRNVCTFHGTM

RVEDS-LCLVMDKCYGSVQSEMQRNEGRLTLEQVLRYGADIARGVVELHA

AGVVCMNLKPSNLLLDANGHAVVSDYGLATILKKPSCWKARPECDSAKIH

SCMECIMLSPHYTAPEAWEPVKKSLNLFWDDGIGISSESDAWSFGCTLVE

MCTGAIPWAGLSAEEIYRAVIKAKKLPPQYASVVGGGIPRELWKMIGECL

QFKPSKRPTFSAMLAIFLRHLQEIPRSPPASPDNGLDKGSVSNVMEPS-P

VPELEVPQENPNHLHRLVSEGDTAGVRDLLAKAASESGSNYLSMLLEAQN

ADGQTALHLACRRGSAELVETILECREANVDVLDKDGDPPLVFALAAGSP

ECVRSLIKRNANVRSRLRDGFGPSVAHVCAYHGQPDCMRELLLAGADPNA

VDDEGESVLHRAIAKKYTDCALVILENGGCRSMAILNPKNLTPLHLCVAT

WNVAVVKRWVEVATSDEIAESIDIPSPIGTALCMAAASKKDHENEGRELV

QILLAAGADPSAQDSQNGRTALHTAAMTNDVDLVKVILGAGVDVNIRNVH

NSIPLHLALARGAKACVGLLLAAGADYNLQDDDGDNAFHIAADTAKMIRE

NLDWLIVMLRNPNADIEVRNHC----------------------GKTLRD

ILEALPREWLSEDLMEALMNRGVHLFPTV---------------------

---------------------------------------------FEVGD

WVKFKRSVTKPKHGWQGAKPKSVGFVQSVPDRDN--LIVSFCSGE--VHV

LANEVIKVIPLDRGQHVQLKEDVKEPRFGWRGQSRDSIGTVLCVD-DDGI

LRVGFPGASRGWKADPAEMERVEEFKVGDWVRIRPTLTSAKHGLGSVTPG

SIGIVYCIRPDSSLLIELSYLPNPWHCEPEEVEHVAPFRIGDRVCVKRSV

AEPRYAWGGETHHSVGRISEIENDGLLIIEIPNRPIPWQADPSDMEKVED

FKVGDWVRVKASVSSPKYGWEDITRTSIGVIHSLEEDGDMGVAFCFRSKP

FSCSVTDVEKVPPFEVGQEIHLMPSVTQPRLGWSNESAATVGKIVRIDMD

GALNVRVTGRQSLWKVSPGDAERLPGFEVGDWVRSKPSLGTRPSYDWNSV

GRESLAVVHSVQDSGYLELACCFRKGKWITHYTDVEKVPSFKVGQYVRFR

TGLVEPRWGWRGAQPESQGVITSIHADGEVRVAFFGLPGLWRGDPSDLEI

EQMFEVGEWVRLNDNANNWKSIGAGSVGVVQGIGYEGDELDRSIFVGFCG

EQEKWVGPSSHLERFDKLSVGQKVRVKQYVKQPRFGWSGHTHASIGTIQA

IDADGKLRIYTPAGSKTWMLDPSEVKVVEEKE-LCIGDWVRVKASISTPT

HHWGEVSHSSIGVVHRMADEDLWVAFCFTERLWLCKAWEMERVRPFKVGD

KVRIRDGLVTPRWGWGMETHASKGQVVGVDANGKLRIKFRWREGRPWIGD

PADLALDED------------

>Medicago_truncatula_KEG

M-----KIPCCSVCQTRYNEEERVPLLLQCGHGFCKECLSRMFSSSSDAN

LTCPRCRHVSTVGNSVQALRKNYAVLSLILSAADSAAAAGGGGGG----D

CDFTDDDEDRDDSEVDDGDDQ------KLDCRKNSRGSQASSSGG----C

APVIEVG-----VHQDLKLVRRI---GEGR--RAGVEMWSAVIGGG----

----RCKHQVAVKKVVLNEGMDLDWMLGKLEDLRRTSMWCRNVCTFHGAM

KVDEG-LCLVMDKCFGSVQSEMLRNEGRLTLEQVLRYGADIARGVVELHA

AGVVCMSLKPSNLLLDANGHAVVSDYGLATILKKPSCWKARPECDSAKIH

SCMECIMLSPHYTAPEAWEPVKKSLNLFWDDGIGISPESDAWSFGCTLVE

MCTGAIPWAGLSAEEIYRQVVKAKKQPPQYASVVGGGIPRELWKMIGECL

QFKPSKRPTFNAMLAIFLRHLQEIPRSPPASPDNDLVKGSVSNVTEAS-P

VPELEIPQD-PNRLHRLVSEGDVTGVRDFLAKAASENESNFISSLLEAQN

ADGQTALHLACRRGSAELVETILDYPEANVDVLDKDGDPPLVFALAAGSH

ECVCSLIKRNANVTSRLRDGLGPSVAHVCAYHGQPDCMRELLLAGADPNA

VDDEGESVLHRAIAKKFTDCALVIVENGGCRSMAISNSKNLTPLHLCVVT

WNVSVVKRWVEVATADEIAEAIDIPSPIGTALCMAAASKKDHESEGRDLV

QILLTAGADPSAQDSQNGRTALHTAAMTNDVDLVQVILAAGVDVNIRNVH

NSIPLHLALARGAKTCVGLLLDAGADCNLQDDDGDNAFHIAAETAKMIRE

NLDWLVVMLLNPDADIEVRNHR----------------------GKTLRD

ILEGLPREWLSEDLMEALVNRGVHLSPIT---------------------

---------------------------------------------FDVLD

WVKFKRTVTEPKHGWQGAKPNSVGFVQSVPGRDNDDLIVSFCSGE--VRV

LTSEIVKLIPLDRGQHVQLKGDVNEPRFGWRGQSRDSIGTVLCVDPEDGI

LRVGFPGASRGWKADPAEMERVEEFKVGDWVRVRPTLTTSKHGLGNVVPG

TIGIVYCIRPDSSLLVELSYVQNPWHCEPEEIEHVPPFRIGDRVCVKRSV

AEPRYAWGGETHHSVGRISEIENDGLLIIEIPNRPIPWQADPSDMEKVED

FKVGDWVRVKASVSSPKYGWEDITRNSIGVIHSLEEDGDMGVAFCFRSKP

FSCSVTDVEKVPPFEVGQEIRVMQSVNQPRLGWSNESPATVGKIVRIDMD

GALNARVTGRQSLWKVSPGDAERLPGFEVGDWVRSKPSLGNRPSYDWNSV

GRESLAVVHSVQDSGYLELACCFRKGKWITHYTDVEKVPSFKVGQYVRFR

PGLAEPRFGWGGAQPESQGIITNIHADGEVRVAFFGLSGLWKGDPSDLQA

EQIFEVGEWVRLKENVNNWKSIGPGSVGVVQGIGYEGGETDRSTFVGFCG

EQEKWVGPSSHLERVDKLIVGQKVRVKQNVKQPRFGWSGHTHASIGTIQA

IDADGKLRIYTPAGSRTWMLDPSEVEVVEEKE-LCIGDWVRVRASVSTPT

PPLG-------------GNDNLWVSFCFVERLWLCKASEMERVRPYKVGD

KVRIRDGLVSPRWGWGMETHASRGHVVGVDANGKLRIRFRWREGRPWIGD

PADIALDEN------------

>Sorghum_bicolor

M-----RVPCCSLCNVRYDEEERTPLLLHCGHGFCRACLSRMLAAAPGAT

LACPRCRHPTAVGNSVSALRKNFPILSLLSASPSSPSFLHSDSG-SSS--

-DGSEDEDD-FFARPSRRP--------------------APAPAAAPPGC

SSF------DLVSHPDLKLARRI---GSGPPGPAGQEVWAGMLSRGGGGG

-GVKRCKHQVAVKRVPLAAGDGLEVVQEEVERLRRASTWCRNVCTFHGAV

RVG-GHLCFVMDRYVGSVQAEMRQNGGRLTLEQILRYGADIARGVAELHA

AGIVCMSIKPSNILLDAHGHAVVSDYGLSAILKNLTSRRVPDDSS-AGI-

---DATLLSPNYTAPEAWGPLKKSLNMFWDSANGISPESDAWSFGCTLVE

MCTGAVPWAGLSAEEICKSVVKEKKPPPQYSRVVGVGLPGELWKMIGECL

QFRASRRPSFQDMLKTFLRHLLDIPRSPPASPENDFTNENLPNGMEPP-T

TSILEMVHDNPNALHHLVCEGDAAGVRDLLAKAASERNGSLIRSLLEAQN

TDGLTALHLACRRGSGELVEAIVAYQE-NVDILDKDEDPPIVFALAAGSP

RCVRALVGRSASINSRLREGLGPTLAHVCAHHGQPECMQELLMAGADPNA

VDGEGESVLHIAVARRYTDCAIVILENGGCRSMGISNSQHKTPLHLCIET

WNTAVVRRWVEIASLEDIAEAIDVPSPVGTALCMAAALKKEHEKEGRELV

RILLAAGADPTAQDDPHCRTALHTAAMIDDVELVKIILEAGVDVNIRNAQ

NTTPLHVALNRGANSCVGLLLAAGANCNIQDDDGDNAFHIAADAAKMIRE

NLTWIAQMLLQPSPAVDVRNHR----------------------GWTLRD

FLERLPREWIYEELMETLEDKGVHLSPTIGNLIRLAPPNFKGRLSWLGSK

GLEDYPTLGGNVKYVEKLAAACTPRARCRGSAGCGWWSTSCGLYTYEVAD

WVKFRRTVTSPAFGWQGAGPRSIGFVQSVVDNDH--LAVSFCTGE--AHV

LTSEVIKVIPLNRGQHVQLKPDVLEPRFGWRGQSRDSIGTVLCVD-DDGI

LRVGFPGASRGWRADPAEIERVEEYKVGNWVRIRPSLTVAVHGMESITPG

SVGIVYSIRPDSSLLLGLCYLSHPWLCEPEEVEHVDPFKIGDQVCVKRSV

AEPRYAWGGETHHSVGKIIDIESDGLLIIDIPNRAAPWQADPSDMEKIEN

FKVGDWIRVKATVPSPKYGWEDVTRNSIGIVHSLQDDGDVGVAFCFRSKL

FLCSVADVEKAQPFEVGEKVHVSPSIPEPRLGWLNETAATIGAISRIDMD

GTLNIKVSGRKSLWKVAPGDAERLSAFEVGDWVRPKPSIGSRPTYDWNSV

GRISIAVVHSIQDSGYLELAGCFRNGKWLTHNTDIEKVQTLKIGQHVRFR

AGISEPRWGWRDANPDSRGIIAGVHADGEVRVAFFGVPGLWRGDPADLEV

ENIFEVGEWVRLRNNVEQWRSLKPGSIGVVHGVGYQGDAWDGTIHVAFCG

EQERWVGPSSQLEGVSKFVVGQRVRIRGCIRHPRFGWSNHSHSSIGTISS

IDADGKLRIHTPAGARAWLIDPAEVEKVEEEEEVCVGDWVKVKDSVATPV

YQWGDVNHNSIGVVHRADDGELWIAFCFCERLWLCKAWEVEKVRPFRQGD

KVRIRPGLVSPRWGWGMETYASKGEVVGVDANGKLRIKFRWRDRL-WIGD

PADIVLDDAHLLTEASNGF--

>Oryza_sativa

M-----RVPCCSLCHVRYDEEERAPLLLHCGHGFCRACLARMLANAAGAV

LACPRCRHPTAVGNSVSALRKNFPILSLLSSSPSSPSFLHSDSGGSSS--

-DGSDDDDDDFFGRPSRRSS------------AEGAGAGAAAPSLQPAGC

ASF------DLASHPDLKLARRI---GSGPPGPAGQEVWSGTLSRGGGGG

-GAKRCKHPVAVKRVPVTAGDVLEGVQEEVERLRRAATWCRNVTTFHGAV

RVG-GHLCFVMDRYAGSVQTEMRQNGGRLTLEQILRYGADIARGVAELHA

AGIVCMSIKPSNILLDTNGHAVVSDYGLSAILKNLTSRRVSDDSNMVGL-

---DATLLSPNYTAPEAWVPLKKSMNLFWDSANGILPESDAWSFGCTLVE

MCTGAVPWAGLSAEEICKSVVKERKPPPQYSRVVGVGLPGELWKMIGDCL

QFKPSRRPSFQDMLKTFLRHLLDIPRSPPASPENDFTNASMPNGMDVP-P

ASVLDMVQDNPNALHHLVCEGDAAGVRNLLAEAASDGNGRLIRSLLEAQN

ADGYTALHLACRRGSAEIVEAIVAYQE-NVDLLDKNENPPIIFAMAAGSP

QCVRALVRRSSDVNSRLREGLGPTLAHVCAHHGQPECMRELLMAGADPNA

VDGEGESILHIAVAKRYTDCAIVLLENGGCRSMGIPNSVNKTPLHLCIET

WNADVVKRWVEVASEEEIAEAIDVPSPVGTALCMAAALKKEHEKEGRELV

RVLLSAGADPTAQDDPHCRTALHTAAMINDAELVKIILEAGVDVNIRNAQ

NTTPLLVALNRGANSCVGLLLAAGANCNLQDDDGDNAFHIAADAAKMIRE

NLSWIVQMLQQPSPAVDVRNHRQVYDVSGVKLNLLQYNVMQSKRGWTLRD

FLERLPREWISEELMETLEEKGVHLTPTI---------------------

---------------------------------------------YEFAD

WVKFRRTVTEPAFGWQGAGPRSIGFVQSVVDHDH--LVVSFCSGE--ARV

LTSEVIKVIPLNRGQHVQLKPDVLEPRFGWRGQSRDSIGTVLCVD-DDGI

LRVGFPGASRGWRADPAEIVRVEEYKVGNWVRIRPSLTVAVHGMESITPG

SVGIVYSIRPDSSLLLGLCYLSSPWLCEPEEVEHVDPFKIGDQVCVKRSV

AEPRYAWGGETHHSVGKIIDIESDGLLIIDIPNRAVHWQADPSDMEKIEK

FKVGDWVRVKATVPSPKYGWEDVTRSSIGVVHSLEEDGDMGVAFCFRSKP

FSCSVADVEKAQPFEVGEKIHVLPSISQPRLGWSNETAATIGAISRIDMD

GTLNVKVSGRNSLWKVAPGDAERLSAFEVGDWVRLKSSIGSRPTYDW-SV

GKISIAVVHSIQDSGYLELAGCFRKGKWLTHNTEIDKVEPLKIGHHVRFR

AGITEPRWGWRDAKPDSRGIIAGVHADGEVRVAFFGVPGLWKGDPADLEI

EQVYEVGEWVRLRNNADDWKSLKPGSIGVVHGIGYEDDVWDGTIHVAFCG

EQERWIGPSSQLEGVSKFVVGQRVRVKLCIRQPRFGWSNHNHSSIGTISS

IDADGKLRIHTPAGARAWLIDPAEVEKVEEEEEVCVGDWVKVKDCVATPT

YQWGDVNHNSIGVVHRAEDGELWVAFCFCERQWLCKRWEVEKVRPFRLGD

RVRIRPGLVTPRWGWGEETYESKGEVVGVDANGKLRIKFRWRDGL-WIGD

PADIVLDDIPSLTEASNGFCS
